# Hydrothermally Synthesized PPy/VO_2_ Nanorod Composites for High-Performance Aqueous Zinc-Ion Battery Cathodes

**DOI:** 10.3390/mi16060705

**Published:** 2025-06-13

**Authors:** Taoyun Zhou, Shilin Li, Dong Xie, Yi Liu, Yun Cheng, Xinyu Li

**Affiliations:** 1 School of Information, Hunan University of Humanities, Science and Technology, Loudi 417099, Chinaxie.dong@139.com (D.X.); yuncheng@huhst.edu.cn (Y.C.); 2 Key Laboratory of Low-Dimensional Structural Physics and Application, Education Department of Guangxi Zhuang Autonomous Region, College of Physics and Electronic Information Engineering, Guilin University of Technology, Guilin 541004, China; lixinyu5260@163.com

**Keywords:** PPy/VO_2_ hybrid nanocomposite, electrochemical performance, zinc-ion battery, cycling stability, high energy density, hierarchical nanostructure

## Abstract

The rapid development of energy storage technologies has led to an increasing demand for high-performance electrode materials that can enhance both the energy density and the cycling stability of batteries. In this study, polypyrrole (PPy) nanorods with partial hollow features are utilized as a conductive and flexible framework for the in situ growth of VO_2_ nanospheres via a simple hydrothermal method, forming a well-defined core–shell PPy/VO_2_ nanocomposite. This hierarchical nanostructure combines the excellent electrical conductivity and mechanical flexibility of PPy with the high theoretical capacity of VO_2_, creating a synergistic effect that significantly enhances the electrochemical performance. The well-integrated interface between PPy and VO_2_ reduces interfacial resistance, promotes efficient electron and ion transport, and improves the overall energy conversion efficiency. Electrochemical testing reveals that the PPy/VO_2_ nanocomposite delivers a high specific capacity of 413 mAh g^−1^ at 100 mA g^−1^ and retains 87.2% of its initial capacity after 1200 cycles, demonstrating exceptional rate capability and long-term cycling stability. This work provides a versatile strategy for designing high-performance cathode materials and highlights the promising potential of PPy/VO_2_ nanocomposites for next-generation high-energy-density aqueous zinc-ion batteries.

## 1. Introduction

The growing global energy demand, along with rising environmental concerns, highlights the urgent need for sustainable and environmentally friendly energy storage technologies [1,2]. Conventional fossil-fuel-based energy sources are increasingly associated with greenhouse gas emissions and environmental degradation, which limit their long-term viability [3]. In response, renewable energy sources such as solar and wind have gained significant traction. However, their intermittency and geographical limitations require the integration of high-performance energy storage devices to ensure grid stability and efficient power management [4,5].

Among various energy storage technologies, rechargeable batteries—particularly lithium-ion batteries (LIBs)—have dominated markets for portable electronics and electric vehicles due to their high energy density and cycle life. However, the limited abundance and uneven global distribution of lithium resources, along with safety risks associated with organic electrolytes and rising costs, hinder their broader application in large-scale energy storage [6,7,8,9,10]. Consequently, alternative battery systems based on earth-abundant, low-cost, and safe materials are being intensively investigated.

Aqueous zinc-ion batteries (ZIBs) have emerged as a promising candidate due to the high volumetric capacity of zinc (5855 mAh cm^−3^), two-electron redox activity, environmental friendliness, and compatibility with water-based electrolytes, which greatly improve safety and reduce manufacturing costs [11,12,13,14,15,16]. Nevertheless, the development of ZIBs is currently constrained by the lack of high-performance cathode materials. Although manganese oxides, Prussian blue analogues, and vanadium-based materials have been explored, many of them suffer from poor rate capability, structural degradation, or low capacity retention during prolonged cycling [17,18,19,20,21].

Among these, vanadium oxides—especially VO_2_—have attracted considerable attention due to their layered or tunnel crystal structures and multiple valence states (V^4+^/V^5+^), which facilitate reversible Zn^2+^ insertion/extraction [22,23,24]. However, VO_2_ alone often exhibits low electrical conductivity, sluggish Zn^2+^ diffusion kinetics, and poor cycling stability due to dissolution in aqueous electrolytes, limiting its practical application in ZIBs. Therefore, enhancing the conductivity, structural integrity, and ion transport pathways of VO_2_-based cathodes is critical to fully realize their potential.

Conductive polymers, such as polypyrrole (PPy), offer a compelling strategy to address these issues. Owing to its π-conjugated structure, intrinsic electrical conductivity, mechanical flexibility, and ability to buffer volume changes, PPy has been widely used in hybrid electrode designs [25,26]. Notably, PPy can form tubular or globular nanostructures that establish interconnected conductive networks and act as protective layers to suppress dissolution of active materials. These features enable improved charge transport and structural stability under electrochemical cycling.

Previous studies have reported PPy-based composites with vanadium oxides, such as V_2_O_5_ or mixed-valence VO_x_, for supercapacitors or other battery systems [25,26]. However, most of these works either focus on non-battery-type devices (e.g., flexible asymmetric supercapacitors), use complex nanowire arrays on substrates, or operate in three-electrode systems, which are less representative of practical full-cell aqueous ZIB configurations. Moreover, the synergistic mechanism between PPy and VO_2_ in true battery environments—particularly regarding interfacial charge transfer, structural stability, and Zn^2+^ diffusion pathways—remains poorly understood.

In this work, we report a facile hydrothermal and self-assembly strategy to synthesize a PPy/VO_2_ hybrid composite, where VO_2_ nanospheres are uniformly anchored and grown in situ on PPy nanorods. Unlike previous approaches, our design yields a free-standing powder with a hierarchical nanostructure that is easily processed into coin cells, enabling direct application in aqueous ZIBs. The composite not only enhances electrical conductivity and structural integrity, but also demonstrates superior rate capability and long-term cycling performance under realistic two-electrode conditions. The improved interfacial engineering and pseudocapacitive kinetics distinguish our material from prior PPy–VO_x_ systems and offer a new route for practical, high-performance zinc-ion energy storage.

Electrochemical results demonstrate that the composite delivers a high specific capacity (413 mAh g^−1^ at 0.1 A g^−1^), excellent rate capability, and remarkable cycling stability (87.2% capacity retention after 1200 cycles). This study not only provides a new route for cathode material design in ZIBs but also contributes to understanding polymer-inorganic hybrid mechanisms for fast and durable energy storage.

## 2. Material and Methods

### 2.1. Materials

All chemicals used in this study are of analytical reagent (AR) grade and were used without further purification. Hydrochloric acid, oxalic acid, methanol, aniline, ethanol, and hydrogen peroxide (30% AR) were purchased from Xilong Chemical Corporation (Shantou, China). Ammonium metavanadate (NH_4_VO_3_) and argon gas (Ar, AR grade) were obtained from Luoen Corporation (Shanghai, China) and Guangzhou Junduo Gas Corporation (Guangzhou, China), respectively. The glass fiber separator (model 1823-047) was supplied by Whatman (Maidstone, UK).

### 2.2. Preparation of PPy Nanorods

To prepare a 2.0 mmol/L methyl orange solution, 0.196 g of methyl orange was dissolved in 300 mL of deionized water. Then, 1 mL (14.4 mmol) of pyrrole monomer was added to the solution, and the mixture was stirred at 0 °C for 1 h. Next, an oxidant solution containing 100 mL of FeCl_3_ was slowly introduced into the reaction system. The polymerization reaction was carried out at 0 °C with continuous mechanical stirring for 24 h. Upon completion of the reaction, the black precipitate was collected by vacuum filtration and washed alternately with deionized water and ethanol six times until the filtrate became colorless. The product was then freeze-dried at −50 °C for 24 h to obtain PPy nanorods.

### 2.3. Synthesis of PPy/VO_2_

In a separate procedure, 100 mL of deionized water was mixed with 3.56 g of oxalic acid and commercial V_2_O_5_ powder, and the mixture was stirred at 60 °C until the solution turned blue. The mixture was then dried at 75 °C for two days and ground into a fine powder for further use.

Next, 60 mg of the prepared V_2_O_5_ powder was added to 60 mL of a methanol–water mixture (methanol/water = 4:1) and stirred for 3 h. Following this, 20 mg of PPy nanorods was added, and the mixture was sonicated for 1 h. The resulting suspension was transferred to an 80 mL autoclave and heated at 200 °C for 24 h.

After the reaction, the product was washed repeatedly with ethanol and deionized water until the filtrate was clear. Finally, the material was dried at 60 °C for 24 h to obtain the PPy/VO_2_ composite.

### 2.4. Electrode Fabrication and Cell Construction

High-purity zinc foil (99.9%) was employed as both the counter and reference electrodes in the electrochemical setup, leveraging its well-defined and reversible Zn/Zn^2+^ redox potential (~0.76 V vs. SHE in 3 M Zn(CF_3_SO_3_)_2_) to ensure stable and reproducible measurements. The working electrode was prepared by thoroughly mixing the active material (PPy/VO_2_ composite or pure VO_2_), Super P carbon, and PVDF binder in a mass ratio of 7:2:1 using N-methyl-2-pyrrolidone (NMP) as the dispersing agent. The resulting slurry was uniformly applied onto stainless-steel mesh current collectors and vacuum-dried at 65 °C for 12 h. The electrode coating was controlled to achieve an active material loading of approximately 2 mg cm^−2^. Coin-type cells (CR2016) were assembled under ambient conditions, incorporating zinc foil electrodes, the prepared cathode, a glass fiber separator (Whatman, UK), and 3 M Zn(CF_3_SO_3_)_2_ aqueous electrolyte to complete the configuration.

### 2.5. Characterization Techniques

The structural, morphological, and chemical properties of the synthesized PPy/VO_2_ composites were comprehensively characterized using various analytical techniques. Powdered samples were prepared according to the specific requirements of each method. To provide a systematic overview of the experimental techniques employed in this study, the instrumentation, key parameters, and analysis software used for structural, morphological, chemical, and electrochemical characterization of PPy/VO_2_ are summarized in Table 1. These methods encompass a comprehensive range of analysis—including phase identification, surface morphology, elemental composition, thermal behavior, surface area measurement, and electrochemical performance—ensuring a thorough understanding of the material properties.

## 3. Results and Discussion

### 3.1. Material Characterization

#### 3.1.1. Scanning Electron Microscopy (SEM) and Energy Dispersive Spectroscopy (EDS) Analysis

**Figure 1:**
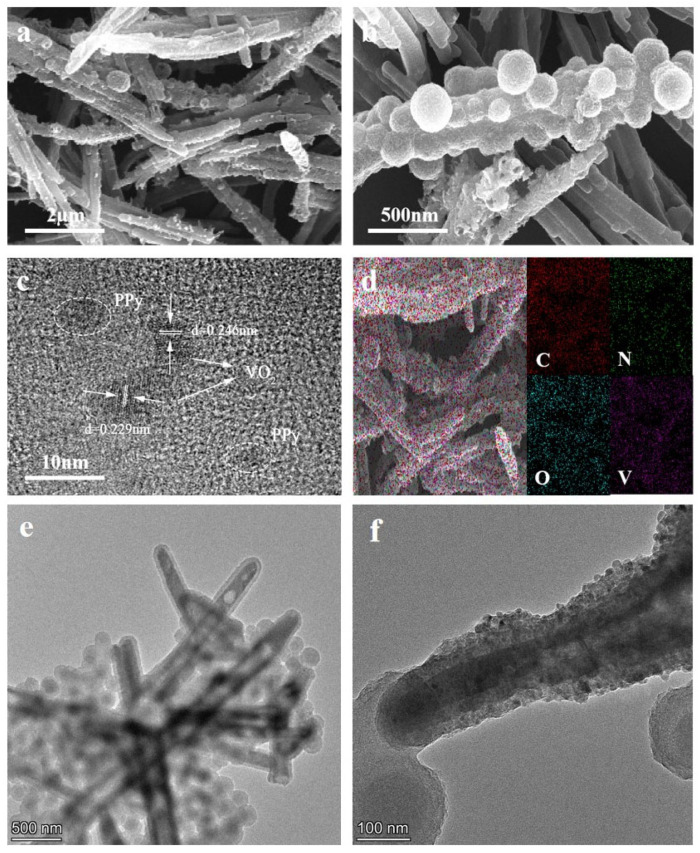
SEM and TEM images of PPy/VO_2_: (**a**,**b**) SEM images of PPy/VO_2_ at different magnifications, showing the morphology and structure, (**c**) HRTEM image of the PPy/VO_2_ composite, (**d**) EDS image of the PPy/VO_2_ composite, and (**e**,**f**) TEM images of the PPy/VO_2_ at different magnifications.

The morphological characteristics of PPy/VO_2_ were investigated using SEM and TEM, as shown in Figure 1. Figure 1a,b presents SEM images of the composite after hydrothermal synthesis.

Figure 1a shows a low-magnification SEM image (scale bar: 2 μm), where PPy/VO_2_ exhibits large-scale rod-like or fibrous structures. These structures, composed of PPy and VO_2_ nanoparticles, appear relatively uniform and well-aligned.

Figure 1b displays a high-magnification SEM image (scale bar: 500 nm), revealing surface features of PPy/VO_2_ at the nanoscale. In this image, certain fibrous structures are observed to be partially decorated with spherical or irregularly shaped nanoparticles, which are tentatively attributed to VO_2_. However, this decoration is not uniformly present on all observed structures, indicating some degree of morphological variation across the sample.

Figure 1c presents a high-resolution TEM (HRTEM) image (scale bar: 10 nm), which offers insights into the fine structural organization at the nanoscale. The observed lattice fringes correspond to interplanar spacings of 0.246 nm and 0.229 nm, matching the (002) and (200) crystallographic planes of VO_2_, respectively [27,28]. The surrounding low-contrast amorphous regions are tentatively assigned to PPy, considering its composition of light elements (C, N, H) and characteristic low electron density.

Figure 1d shows the elemental distribution of PPy/VO_2_ obtained via EDS. Elemental mapping reveals the presence and distribution of carbon (C), nitrogen (N), oxygen (O), and vanadium (V). The homogeneous distribution of C and N elements indicates the uniform dispersion of PPy, while O and V are evenly distributed throughout the sample, confirming the presence of VO_2_. These findings demonstrate the successful integration of PPy and VO_2_.

Figure 1e,f validates the formation of PPy/VO_2_ nanorods with a core–shell architecture. In Figure 1e, the nanorods exhibit partial surface decoration with VO_2_ nanospheres, indicating some degree of morphological non-uniformity. In contrast, Figure 1f displays a single nanorod uniformly coated with nanospheres, confirming the successful formation of a core–shell structure at the individual level. Although minor inconsistencies in surface coverage are observed, the overall composite maintains a robust architecture, which is advantageous for improving structural integrity and electrochemical performance.

#### 3.1.2. X-Ray Diffraction (XRD) and Raman Spectroscopy Analysis

**Figure 2:**
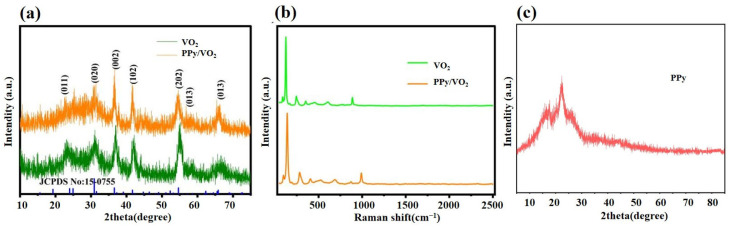
Characterization of PPy/VO_2_ and VO_2_ samples: (**a**) XRD patterns of PPy/VO_2_ and VO_2_, (**b**) Raman spectra of PPy/VO_2_ and VO_2_, and (**c**) XRD patterns of PPy.

XRD analysis is performed on the synthesized VO_2_, PPy/VO_2_ composites, and PPy, and the results are shown in Figure 2.

Figure 2a presents the XRD patterns of VO_2_ and PPy/VO_2_. The characteristic diffraction peaks of VO_2_ in PPy/VO_2_ are consistent with the standard pattern for monoclinic VO_2_ (JCPDS No. 15-0755), indicating that the incorporation of PPy does not alter the crystalline phase of VO_2_.

Specifically, diffraction peaks at 24.92°, 30.93°, 36.57°, 41.66°, 39.12°, and 54.62° correspond to the (110), (111), (002), (200), (102), and (202) planes, respectively, confirming the retention of the monoclinic phase with lattice parameters a = 4.5968 Å, b = 5.6844 Å, and c = 4.9133 Å [29].

Notably, the PPy/VO_2_ composite exhibits broader diffraction peaks in the 25–30° range, accompanied by increased peak intensity. This phenomenon may be attributed to the interaction of PPy with the VO_2_ matrix.

Figure 2b shows the Raman spectra of VO_2_ and PPy/VO_2_, in which the Raman spectrum of PPy/VO_2_ does not show significant changes compared to VO_2_. This suggests that, while the introduction of PPy may lead to changes in the lattice or local structure, it does not induce significant chemical structural changes [30]. The major Raman peaks of both materials remain consistent within the range of 500 cm^−1^ to 2500 cm^−1^, indicating that the crystal structure of VO_2_ still dominates in the composite.

Figure 2c shows the XRD patterns of PPy, which displays a broad diffraction peak around 20–30°, indicating the amorphous or low-crystalline nature of pure PPy. Compared to this, PPy/VO_2_ exhibits distinct crystalline VO_2_ peaks and significantly enhanced electrochemical performance, including a much higher specific capacity and structural stability.

#### 3.1.3. Thermogravimetric (TGA) Analysis

**Figure 3:**
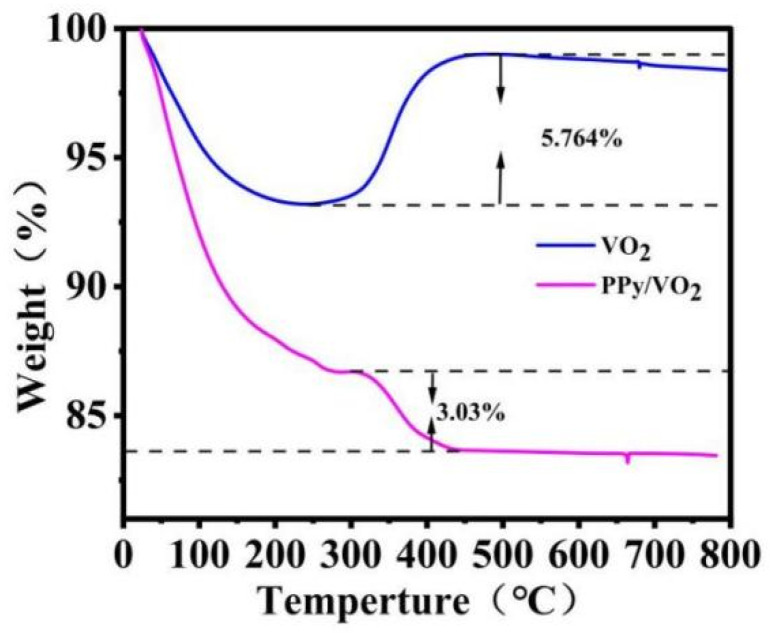
TGA curves of PPy/VO_2_ at different temperatures.

TGA analysis provides an intuitive view of the proportions of the components in the composite material and its thermal stability. Figure 3 shows the TGA curves of PPy/VO_2_ and VO_2_.

In the temperature range from 0 °C to 300 °C, both VO_2_ and PPy/VO_2_ samples exhibit a sharp weight loss, which is primarily attributed to the evaporation of physically adsorbed and interlayer water, as well as the possible removal of volatile surface species. This indicates that moisture and low-boiling-point substances are gradually expelled from the materials at lower temperatures.

Between 300 °C and 800 °C, a distinct divergence is observed between the two curves. For the pure VO_2_ sample, a mass gain of approximately 5.764% is recorded. This increase is ascribed to the oxidation of VO_2_ to V_2_O_5_, a well-known transformation that occurs in this temperature range [31]. The formation of V_2_O_5_ introduces additional oxygen into the lattice, leading to a net mass increase, which then gradually stabilizes at higher temperatures.

In contrast, the PPy/VO_2_ composite exhibits more complex thermal behavior in the same temperature region. Around 300 °C, a transition point is observed where the mass of the composite begins to decline. This weight loss is attributed to the thermal decomposition and oxidative degradation of the PPy component, which undergoes carbonization and combustion in the oxidative atmosphere [31].

The total weight loss of the composite between 300 °C and 800 °C is approximately 3.03%, which is initially used as a rough estimate of the PPy content. This value reflects the combined effects of PPy decomposition and VO_2_ oxidation, including the potential mass gain from the VO_2_ → V_2_O_5_ conversion. Therefore, the actual PPy content is likely slightly higher than 3.03%.

Nevertheless, the TGA data still indicate that the PPy content in the composite is relatively low, which is consistent with the minor structural modifications observed in the XRD and Raman spectra, and provides a reasonable basis for interpreting the interfacial role of PPy in the composite.

#### 3.1.4. Nitrogen Adsorption/Desorption Test

**Figure 4:**
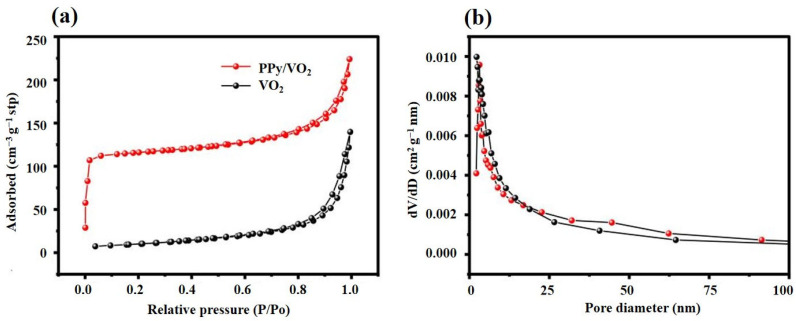
Nitrogen adsorption/desorption isotherms and pore size distribution of PPy/VO_2_ and VO_2_ samples: (**a**) Nitrogen adsorption/desorption isotherms, and (**b**) BJH pore size distribution curve.

To study the pore size distribution and pore structure characteristics of the synthesized materials, nitrogen adsorption-desorption tests are performed on both VO_2_ and PPy/VO_2_ samples, as shown in Figure 4.

Figure 4a shows the nitrogen adsorption/desorption isotherms of VO_2_ and PPy/VO_2_ measured at 77 K. Both materials exhibit isotherms resembling type IV characteristics according to the IUPAC classification [32], with clear hysteresis loops appearing in the high relative pressure region (P/P_0_ > 0.8), indicative of mesoporous structures. Compared to pristine VO_2_, PPy/VO_2_ demonstrates a significantly higher adsorption capacity across the entire pressure range, suggesting an increase in both pore volume and specific surface area. The BET surface area (SSA) increases from 139.75 m^2^/g for VO_2_ to 224.04 m^2^/g for PPy/VO_2_, representing an improvement of approximately 60%. This enhanced surface area offers more electrochemically active sites, facilitating improved Zn^2+^ adsorption, electrolyte accessibility, and interfacial charge transfer.

However, it is noteworthy that PPy/VO_2_ exhibits a sharp increase in nitrogen uptake at very low relative pressures (P/P_0_ < 0.02), which is typically associated with micropore filling or strong adsorbate–adsorbent interactions [32]. Such behavior is uncommon in PPy/VO_2_ systems. This unexpected feature raises the possibility of instrumental artifacts (e.g., incomplete degassing) or the presence of minor micropores not accounted for in the synthesis design.

Due to the limitations of the BJH method in accurately resolving micropores, further confirmation using DFT-based pore size distribution analysis or additional degassing and repeat measurements would be necessary to fully validate the porosity profile. This refinement will be addressed in future work to ensure the reliability of the data.

As shown in Figure 4b, the pore size distribution derived from the BJH method reveals bimodal mesoporosity, with dominant pore sizes centered at ~2.85 nm and ~22.63 nm. This multiscale pore structure enhances electrolyte infiltration and ion diffusion, while the hierarchical porosity contributes to efficient charge transport, both of which are beneficial for boosting electrochemical performance [32].

#### 3.1.5. X-Ray Photoelectron Spectroscopy (XPS) Analysis

**Figure 5:**
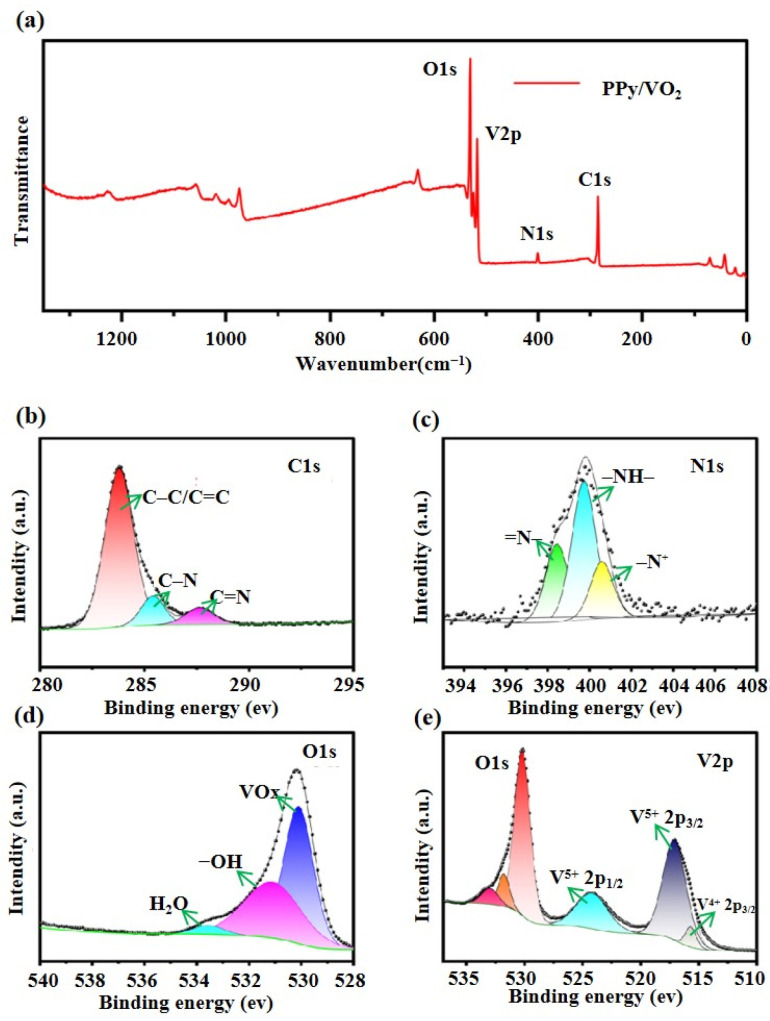
XPS analysis of PPy/VO_2_: (**a**) survey spectrum; (**b**) high-resolution C 1s spectrum; (**c**) high-resolution N 1s spectrum; (**d**) high-resolution O 1s spectrum; and (**e**) high-resolution V 2p spectrum.

To further investigate the surface elemental composition and chemical states of PPy/VO_2_, XPS analysis is conducted, as presented in Figure 5.

Figure 5a shows the survey spectrum of the PPy/VO_2_ sample, clearly indicating the presence of characteristic peaks corresponding to O 1s, V 2p, N 1s, and C 1s, which are the main constituents of the material.

Figure 5b presents the high-resolution C 1s spectrum, which exhibits three distinct peaks at approximately 283.2 eV, 285.6 eV, and 287.4 eV. These are attributed to C–C/C=C, C–N, and C=N bonds, respectively, confirming the carbon bonding configurations within the PPy backbone and verifying the successful incorporation of PPy [33].

Figure 5c shows the N 1s spectrum, which reveals three nitrogen species with binding energies at 398.4 eV, 399.68 eV, and 400.68 eV. These peaks correspond to quinoid (=N–), benzenoid (–NH–), and positively charged quaternary nitrogen (–N⁺), reflecting the diverse nitrogen bonding configurations within the PPy framework.

Figure 5d displays the high-resolution O 1s spectrum, with three main peaks observed at 530.1 eV, 531.6 eV, and 533.7 eV. These correspond to lattice oxygen in VO_x_, hydroxyl groups (–OH) in the PPy chains, and adsorbed water (H_2_O), respectively. Notably, the prominent peak at 530.1 eV suggests the presence of partially oxidized V_2_O_5_ components on the VO_2_ surface [25].

Figure 5e depicts the V 2p spectrum, where the peak at 524.18 eV is attributed to the V 2p_1_/_2_ orbital of V^5+^. Peaks at 515.18 eV and 517.18 eV correspond to the V 2p_3_/_2_ orbitals of V^4+^ and V^5+^, respectively, indicating the coexistence of vanadium in both +4 and +5 oxidation states [26]. The presence of V^5+^ may result from residual V_2_O_5_ on the VO_2_ surface or partial oxidation of VO_2_ during synthesis.

### 3.2. Electrochemical Performance

To evaluate the Zn^2^⁺ storage capability of the synthesized material as a cathode for AZIBs, CR2016-type coin cells are assembled under ambient conditions, as detailed in the Materials and Methods section.

#### 3.2.1. Cyclic Voltammetry (CV) Test

**Figure 6:**
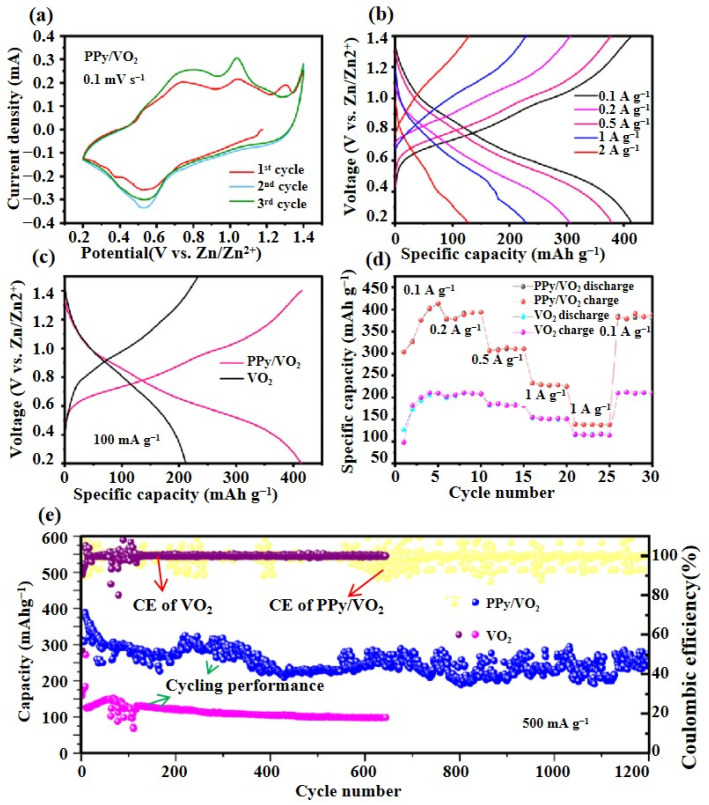
Electrochemical performance of PPy/VO_2_: (**a**) CV curves of PPy/VO_2_ at a scan rate of 0.1 mV s^−1^ in the voltage range of 0.2–1.4 V during the initial three cycles; (**b**) GCD curves at different current densities (0.1–2 A g^−1^); (**c**) comparison of the discharge capacities of pristine VO_2_ and PPy/VO_2_ at 100 mA g^−1^; (**d**) rate capability of PPy/VO_2_ at various current densities and its capacity recovery; and (**e**) cycling performance and Coulombic efficiency of PPy/VO_2_ and VO_2_ electrodes at 0.5 A g^−1^.

Figure 6 presents the electrochemical performance of PPy/VO_2_, including cyclic voltammetry, rate capability, capacity comparison, rate performance, and cycling stability.

Figure 6a displays the CV curves of PPy/VO_2_ at a scan rate of 0.1 mV s^−1^ within the voltage window of 0.2–1.4 V during the initial three cycles. Three distinct redox peaks are observed, indicating the Zn^2+^ storage mechanism. The cathodic peak at approximately 0.52 V corresponds to the intercalation of Zn^2+^ ions, while the anodic peaks at 0.8 V and 1.0 V are associated with the deintercalation process.

As shown in Figure 6b, the GCD profiles of PPy/VO_2_ at different current densities reveal minimal variation in shape, even when the current density increases from 0.1 A g^−1^ to 2 A g^−1^. This indicates favorable structural robustness and fast reaction kinetics. Notably, the distinct voltage plateaus are retained at higher current densities, confirming the superior rate performance of the PPy/VO_2_ system.

A comparison of the charge–discharge performance between pristine VO_2_ and PPy/VO_2_ at a current density of 100 mA g^−1^ is presented in Figure 6c. PPy/VO_2_ exhibits a high discharge capacity of 413 mAh g^−1^, significantly surpassing that of VO_2_ (210 mAh g^−1^). This enhancement is attributed to the conductive network and buffering structure provided by PPy [26].

Figure 6d illustrates the rate capability of PPy/VO_2_ at various current densities (0.1, 0.2, 0.5, 1, and 2 A g^−1^), yielding average discharge capacities of 413.87, 379.56, 306.47, 225.37, and 139.2 mAh g^−1^, respectively. Importantly, when the current density returns to 0.1 A g^−1^, the capacity recovers to 391.04 mAh g^−1^, demonstrating outstanding rate capability and structural reversibility.

Long-term cycling performance is shown in Figure 6e. After 1200 cycles at a current density of 0.5 A g^−1^, PPy/VO_2_ retains 87.2% of its initial capacity, which is significantly higher than that of pristine VO_2_, whose capacity retention drops to 64.3% after 600 cycles.

However, noticeable fluctuations in the capacity profile are observed during long-term cycling. These variations may stem from several factors [29,30]. One plausible cause is electrode instability resulting from repeated volume changes during Zn^2+^ intercalation/deintercalation. Another possible explanation is partial dissolution of vanadium species into the aqueous electrolyte, a well-documented issue in vanadium-based cathodes that can alter the redox environment and introduce cycling instability. Additionally, side reactions such as electrolyte decomposition or the formation/dissolution of intermediate zinc-vanadium complexes may also contribute to the observed fluctuations.

Despite these variations, the coulombic efficiency (CE) remains consistently close to 100% throughout all 1200 cycles, suggesting that the charge/discharge processes are highly reversible and that no significant parasitic reactions dominate [26].

#### 3.2.2. Electrochemical Kinetics Analysis

**Figure 7:**
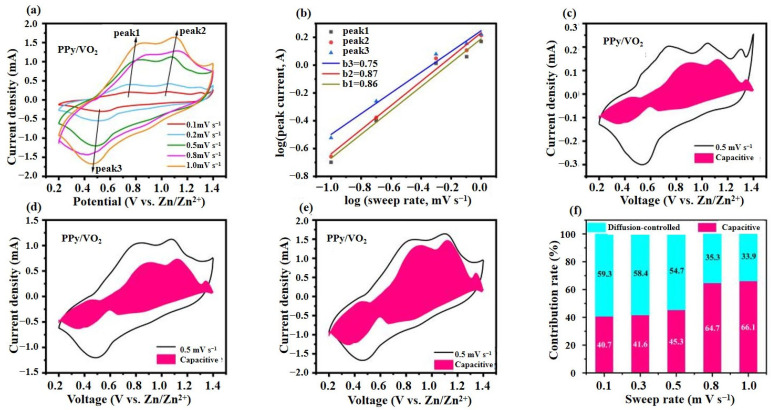
Electrochemical kinetics analysis of PPy/VO_2_. (**a**) CV curves of PPy/VO_2_ at various scan rates; (**b**) log–log plots of peak current (log(i)) versus scan rate (log(v)) derived from the CV curves; (**c**–**e**) separation of capacitive and diffusion-controlled current contributions at scan rates of 0.1, 0.5, and 1.0 mV s^−1^, respectively; and (**f**) variation in the proportion of capacitive contribution with increasing scan rate.

Figure 7 systematically illustrates the electrochemical kinetics behavior of PPy/VO_2_, providing insight into the charge storage mechanism that underpins its excellent rate capability.

As shown in Figure 7a, the CV curves of PPy/VO_2_ recorded at scan rates ranging from 0.1 to 1.0 mV s^−1^ consistently exhibit three well-defined redox peak pairs, which are indicative of multi-step Zn^2+^ insertion/extraction processes and favorable electrochemical reversibility. With increasing scan rate, the anodic peaks gradually shift toward higher potentials, and the cathodic peaks shift toward lower potentials, which reflects increasing polarization and kinetic limitations in the redox reactions—an expected behavior in diffusion-controlled systems [34].

At a scan rate of 0.8 mV s^−1^, a significant peak shift is observed. This shift occurs because, as the scan rate increases, the ion diffusion and charge transfer rates in the electrochemical reactions are limited, leading to polarization at the electrode surface. This polarization causes the redox peaks to shift, with the anodic peak moving to higher potentials and the cathodic peak moving to lower potentials. Such behavior is typical in diffusion-controlled systems [35].

To further investigate the energy storage mechanism, a classical electrochemical kinetics model is employed for quantitative analysis [16,36].

The relationship between scan rate (*v*) and peak current (*i*) can be described as follows:

(1) $$i=av^{b}$$

The logarithmic form of Equation (1) is as follows:

(2) logi=blogv+log(a)

The calculation of the slope *b* provides a qualitative analysis of the electrochemical kinetics. Typically, the value of *b* ranges between 0.5 and 1.0. When *b* = 0.5, the process is diffusion-controlled, whereas *b* = 1 indicates a surface capacitive process.

By performing a linear fitting of the logarithmic relationship between peak current and scan rate for each redox peak in CV curves (Figure 7b), the *b*-values corresponding to different peaks are determined to be 0.75, 0.87, and 0.86, respectively. These *b*-values are significantly greater than 0.5 and approach 1.0, indicating that the energy storage process of PPy/VO_2_ is primarily governed by surface-controlled pseudocapacitive behavior rather than the traditional diffusion-controlled intercalation mechanism. This result suggests that the PPy/VO_2_ material enables fast and reversible Zn^2+^ insertion/extraction, exhibiting a rapid electrochemical kinetics response.

According to the semi-empirical method proposed by Dunn et al. [37,38], the total CV current *i* can be divided into two components: surface capacitive current ($k_{1}v$) and diffusion-controlled current ($k_{2}\sqrt{v}$), both of which vary with scan rate *v*.

Equation (3) is used to characterize and differentiate the contributions of surface capacitive and diffusion-controlled redox processes:

(3) $$i=k_{1}v+k_{2}\sqrt{v}$$

where *i* (A) represents response current, *v* (mV·s^−1^) denotes scan rate, and *k*_1_ and *k*_2_ are constants. For a given potential, the surface capacity and diffusion-controlled contribution can be calculated by fitting the linear relationship according to Equation (4).

Divide both sides of Equation (3) by $\sqrt{v}$:

(4) iv=k1v+k2

Equation (4) determines the proportion of surface capacity contribution at different scan rates by utilizing the integral area ratio between the surface capacity response current and the total current.

Figure 7c–e further analyzes the current responses at different scan rates, separating capacitive contribution (highlighted in pink) from the diffusion-controlled component. Specifically, at a scan rate of 0.5 mV s^−1^, the capacitive contribution accounts for 45.3% of the total capacity (Figure 7d), and this value increases to 66.1% when the scan rate is elevated to 1.0 mV s^−1^ (Figure 7e).

The bar chart in Figure 7f clearly illustrates that the capacitive contribution gradually increases from 40.7% at 0.1 mV s^−1^ to 66.1% at 1.0 mV s^−1^, indicating that under fast scan conditions, PPy/VO_2_ predominantly relies on a surface-controlled pseudocapacitive mechanism for energy storage.

In summary, PPy/VO_2_ exhibits a distinct surface capacitance-dominated behavior, with its Zn^2+^ storage process primarily governed by rapid and reversible pseudocapacitive reactions. This significantly enhances the energy output efficiency and reaction kinetics of the material at high charge/discharge rates.

#### 3.2.3. Electrochemical Impedance Spectroscopy (EIS) Analysis

**Figure 8:**
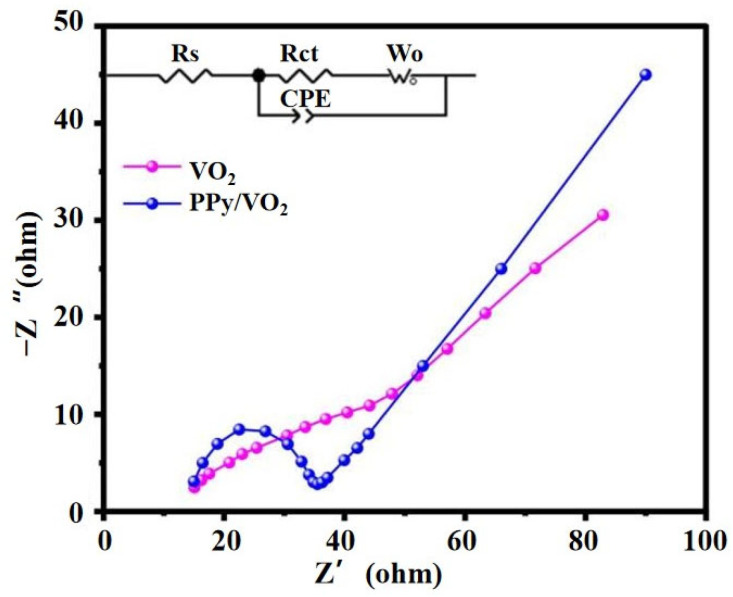
Impedance test of PPy/VO_2_ and VO_2_.

The electrochemical impedance spectroscopy (EIS) results for PPy/VO_2_ and pristine VO_2_ are presented in Figure 8. The distinct differences in their Nyquist plots under identical conditions provide insights into their respective electron transport and ion diffusion capabilities. Each Nyquist plot consists of a semicircle in the high-frequency region and a straight line in the low-frequency region [30,32]. The high-frequency semicircle corresponds to the charge transfer process, while the low-frequency inclined line is attributed to ion diffusion.

The inset in the top-left corner of Figure 8 shows the equivalent circuit model used for fitting, comprising the series resistance (Rs), charge transfer resistance (Rct), constant phase element (CPE), and Warburg diffusion impedance (W_0_).

According to the fitted data, the Rct of PPy/VO_2_ is 16.42 Ω, which is significantly lower than that of VO_2_ (45.23 Ω). As a conductive polymer, PPy effectively forms a three-dimensional conductive network, promoting interfacial electron transport and significantly reducing the energy barrier for charge transfer. This structural feature is crucial for improving the charge transport kinetics of the electrode material [25,26].

The updated EIS fitting results (Table 2) further support this conclusion. Compared to pure VO_2_, PPy/VO_2_ demonstrates significantly lower Rct (16.4 Ω vs. 45.2 Ω), reduced Warburg impedance (Wo-R and Wo-T), and improved CPE parameters that are closer to ideal capacitive behavior.

### 3.3. Performance Analysis

To comprehensively evaluate the electrochemical performance of PPy/VO_2_ developed in this work, we conducted a multidimensional comparison with several representative VO_2_-based composite electrodes, focusing on specific capacity, cycling stability, rate adaptability, and structural–electrolyte compatibility (Table 3) [25,26,39,40].

(1) Specific capacity at comparable current densities

Among the reported materials, PPy/VO_2_ delivers a remarkable specific capacity of 413 mAh g^−1^ at 0.1 A g^−1^, outperforming V^5+^-VO_2_@PPy-180 (314.2 mAh g^−1^ at 0.5 A g^−1^) [25] and MnO_2_/PPy (205.2 mAh g^−1^ at 0.1 A g^−1^) [39]. Although other VO_2_-based hybrid systems—such as VO_2_@PPy nanowire arrays [26] and VO_2_(D)/PPy/g-C_3_N_4_ composites [39]—also exhibit promising capacitive characteristics, their performance is primarily evaluated under non-battery conditions (e.g., fiber-type supercapacitors or three-electrode CV setups), limiting their applicability to practical full-cell ZIBs environments. In contrast, the PPy/VO_2_ composite is tested in a two-electrode configuration with 3 M Zn(CF_3_SO_3_)_2_ electrolyte, rendering the reported capacity directly relevant to real-world aqueous battery systems.

(2) Cycling stability

PPy/VO_2_ exhibits excellent cycling durability, retaining 87.2% of its initial capacity after 1200 cycles at 0.1 A g^−1^. This is comparable to the stability of V^5+^-VO_2_@PPy-180 (95.99% after 1500 cycles at 0.5 A g^−1^) and significantly superior to MnO_2_/PPy (75% after 1000 cycles) [39].

(3) Rate performance and high-rate adaptability

While many VO_2_-based electrodes demonstrate high capacities at moderate current densities, few studies report detailed rate performance across a wide range of current loads. In this work, PPy/VO_2_ maintains excellent redox reversibility and structural stability under increasing rates, delivering 139.2 mAh g^−1^ at 2 A g^−1^ and recovering to 391.0 mAh g^−1^ when the current density returns to 0.1 A g^−1^.

(4) Interfacial charge transfer and electrolyte compatibility

EIS reveals that PPy/VO_2_ exhibits a significantly reduced Rct (16.42 Ω) compared to pure VO_2_ (45.23 Ω), indicating the formation of an efficient conductive interface between PPy and VO_2_. Furthermore, the use of Zn(CF_3_SO_3_)_2_ as the sole electrolyte eliminates interference from Mn^2+^-based buffering effects, as observed in MnO_2_/PPy systems [36], ensuring that the performance enhancement stems from the intrinsic properties of the composite rather than electrolyte composition.

(5) Scalability and practicality electrode processing

Unlike VO_2_@PPy nanowire arrays grown on carbon nanorod (CNT) fibers [26], which require intricate substrate templating and microfabrication steps, PPy/VO_2_ is synthesized via a simple, scalable hydrothermal self-assembly method. The resulting free-standing powder can be readily incorporated into standard slurry-cast electrodes, enhancing the material’s compatibility with existing manufacturing infrastructure and its potential for commercial application.

## 4. Conclusions

In this study, a PPy/VO_2_ nanocomposite is successfully synthesized via a simple combination of hydrothermal reaction and self-assembly, wherein VO_2_ nanospheres are grown in situ on polypyrrole (PPy) nanorods, forming a well-defined core–shell architecture. The resulting composite is applied as a cathode material for aqueous ZIBs. Experimental results demonstrate that the composite offers significant advantages in structural design and interfacial optimization. The PPy nanorods provide a stable and conductive framework, while the uniform dispersion and strong anchoring of VO_2_ nanospheres enhance ionic and electronic transport, reduce interfacial resistance, and improve the overall energy conversion efficiency of the battery.

Electrochemical performance evaluations show that PPy/VO_2_ exhibits a high specific capacity, excellent rate capability, and superior long-term cycling stability. It achieves a discharge capacity of 413 mAh g^−1^ at a current density of 100 mA g^−1^ and maintains 87.2% of its initial capacity after 1200 cycles, highlighting its considerable potential for high-performance energy storage applications. These findings not only provide a new route for cathode material optimization in ZIBs but also open avenues for designing polymer-inorganic hybrid systems with improved interface engineering and pseudocapacitive behavior in next-generation aqueous batteries.

## Figures and Tables

**Table 1:** Summary of characterization techniques, instruments, and operating conditions.

Technique	Instrument & Manufacturer	Key Parameters	Software
XRD	Rigaku D/MAX-2500 (Rigaku, Tokyo, Japan)	Cu Kα (λ = 1.5406 Å); 40 kV, 30 mA; 5°/min; 0.02° step Cu Kα (λ = 1.5406 Å), 40 kV, 30 mA, 2θ: 10~70°, 5°/min, 0.02° step	MDI Jade 6.5
SEM & EDS	Hitachi S-4800 (Hitachi, Tokyo, Japan) + Oxford EDS (Oxford, UK)	5–20 kV accelerating voltage	Quartz PCI 8.0, INCA 5.05
TEM	JEOL JEM-2100 (JEOL, Tokyo, Japan)	200 kV accelerating voltage	Digital Micrograph 3.30.2004
XPS	Thermo ESCALAB 250Xi (Thermo Fisher, Waltham, MA, USA)	Al Kα (1486.6 eV), pass energy: 20 eV, resolution: 0.5 eV, base pressure < 5 × 10^−9^ mbar	Thermo Avantage 5.991
Raman Spectroscopy	LabRAM HR Evolution (Horiba, Kyoto, Japan)	Excitation: 512 nm, room temperature	LabSpec 6.5
BET/BJH	ASAP 2460 (Micromeritics, Norcross, GA, USA)	−196 °C, N_2_ adsorption–desorption, BET for SSA, BJH for pore size	MicroActive 6.07
TGA/DTA	SDT Q600 (TA Instruments, New Castle, DE, USA)	Air atmosphere, RT–800 °C, heating rate: 10 °C/min	Universal Analysis 5.5
GCD	Neware CT4008 (Neware, Shenzhen, China)	Voltage range: 0.2~1.4 V (vs. Zn/Zn^2+^), room temperature	Neware BTSDA 7.6
CV, EIS	CHI760E (Chenhua Instruments, Shanghai, China)	CV: 0.1–1.0 mV/s; EIS: 100 kHz–0.01 Hz, 5 mV AC amplitude, two-electrode system, room temperature	CHI Software 14.01

**Table 2:** Analytical data of electrochemical impedance spectroscopy (EIS).

Sample	Rs (Ω)	Rct (Ω)	CPE-T	CPE-P	Wo-R (Ω)	Wo-T (s)	Wo-P
PPy/VO_2_	18.0	16.4	2.50 × 10^−5^	0.88	6.0	55.0	0.450
VO_2_	35.0	45.2	1.50 × 10^−5^	0.85	10.0	70.0	0.450

**Table 3:** Comparative analysis of electrochemical performance of selected VO_2_-based electrode materials for energy storage applications.

Materials	Current Density	Discharge Capacity	Cycles	Capacity Retention	Electrolyte	System Type	Ref.
V5^+^-VO_2_@PPy-180	0.5 A/g	314.2 mAh/g	1500	95.99%	3 M Zn(CF_3_SO_3_)_2_	Aqueous ZIB	[25]
VO_2_@PPy NW Arrays	—	—	4000	88.9%	—	Supercapacitor (FASC)	[26]
VO_2_(D)/PPy/g-C_3_N_4_	0.5 A/g	—	2000	99.71%	1 M H_2_SO_4_(3-electrode system)	3-electrode system (CV only)	[39]
MnO_2_/PPy	0.1 A/g	205.2 mAh/g	1000	75%	2 M ZnSO_4_ + 0.1 M MnSO_4_	Aqueous ZIB	[40]
PPy/VO_2_	0.1 A/g	413 mAh/g	1200	87.2%	3 M Zn(CF_3_SO_3_)_2_	Aqueous ZIB	This work

## Data Availability

The data used to support the findings of this study are included within the article.
